# Evaluation of health and environmental risks for juvenile tilapia (*Oreochromis niloticus*) exposed to florfenicol

**DOI:** 10.1016/j.heliyon.2020.e05716

**Published:** 2020-12-16

**Authors:** Letícia Sayuri Shiroma, Michelly Pereira Soares, Israel Luz Cardoso, Marcia Mayumi Ishikawa, Claudio Martin Jonsson, Sonia Claudia Nascimento Queiroz

**Affiliations:** a Institute of Chemistry, University of Campinas, POB 6154, 13083-970, Campinas, SP, Brazil; b Joint Graduate Program in Physiological Sciences, Federal University of São Carlos -UFSCar/São Paulo State University, UNESP Campus Araraquara, 14801-903, Araraquara, SP, Brazil; c Embrapa Environment, Brazilian Agricultural Research Corporation (EMBRAPA), Rod. SP 340, Km 127,5, Caixa Postal 69, CEP: 13820-000, Jaguariúna, SP, Brazil

**Keywords:** Antibiotics, Hematological variables, Oxidative stress, Aquaculture, Agricultural water management, Environmental science, Environmental risk assessment, Environmental toxicology, Toxicology

## Abstract

Intensive fish cultivation has a high incidence of infection, which is often controlled by administering antibiotics. Florfenicol (FF) is one of the two antimicrobial drugs permitted for aquaculture in Brazil. Due to their intensive use, potentially harmful effects on aquatic organisms are of great concern. In this sense, we investigated whether the presence of FF in cultivation water could change the health parameters of Nile tilapia. For this, we evaluated hemoglobin, hematocrit, mean corpuscular hemoglobin (MCHC) concentration, mean corpuscular volume (MCV), total plasma protein (TPP), number of circulating red blood cells and leukocytes, as lipid peroxidation levels, catalase activity and glutathione S-transferase activity of fish exposed to 11.72 mg L^−1^ of FF in water for 48 h. The fish were divided into two groups: Nile tilapia in water with FF or without FF (control). Exposure to FF in cultivation water for a short period didn't change the hematological variables analyzed, but caused changes in liver ROS (Reactive oxygen species) markers of the Nile tilapia, which was revealed by lipid peroxidation levels, catalase activity, and glutathione S-transferase. The 48h exposure period was enough to induce oxidative stress in hepatocytes, causing cellular oxidative damage. Therefore, the antibiotic florfenicol may cause toxicity to organisms and aquatic ecosystems, even at a sublethal concentrations near 1/100 LC50-48h for fish species.

## 1 Introduction

According to the FAO, aquaculture has grown at an annual average rate of 5.3% over the past 10 years (FAO, 2020). This has occurred because fish are important sources of protein and are rich in vitamins and omega-3s (Jackson et al., 2019). Among the cultivated species, tilapia is the most produced fish in Brazil, corresponding to over half of the country's production with great economic interest (Jonsson et al., 2019; Piscicultura, 2018), and representing 8.3% of the total world fish production, following carp fishes only (FAO, 2020).

To supply this demand for fish production, intensive farming has emerged. However, the high density of fish can cause infections in this system due to poor water quality, insufficient fish nutrition, ecological changes and environmental degradation (Pulkkinen et al., 2010).

Antibiotics are compounds that are administrated to control these fish diseases. In Brazil, two antimicrobials are used in aquaculture: florfenicol and oxytetracycline (Andrade et al., 2017; Carraschi et al., 2011; Nunes et al., 2018). Florfenicol has been used in Brazil since 2007 (Orlando et al., 2016) and is one of the most widely used in aquaculture throughout the world (Norambuena et al., 2013).

Florfenicol is a veterinary drug that has been widely used to treat infections in fish due to its broad spectrum of action, which acts against both Gram-positive and negative bacteria such as *Aeromonas salmonicida*, *A. hydrophyla*, *Flavobacterium psychrophilum*, *Yersinia ruckeri* and *Vibrio anguillarum* (Carraschi et al., 2011). The mechanism of action of this antibiotic is based on its ability to inhibit protein synthesis by binding to the 50S fraction of the bacterial ribosome (Orlando et al., 2016).

Despite the benefits of using antibiotics such as florfenicol in fish production, problems such as immunosuppression, growth retardation, development of resistant bacterial strains, and environmental problems such as drug residues, have been associated with their use (Saglam and Yonar, 2009). Veterinary drugs can be leached from feed into the water, and can consequently incorporate into aquatic organisms’ tissues through water exposure (Barreto et al., 2018; Rigos et al., 1999). In this sense, florfenicol has been frequently detected in aquatic ecosystems, typically at very low levels (μg L^−1^ or ng L^−1^) (Sørensen and Elbæk 2004; Gordon et al., 2007). The negative impacts of excessive antibiotic residues have been well documented, thus making fish farming concerning regarding the development of microbials that are resistant to antibiotics, which risks both human and environmental health associated with antibiotic use (Binh et al., 2018).

In pisciculture, oral medication administration is the most commonly used method (Gonc et al., 2007). Florfenicol leaching in water is more pronounced at higher temperatures, progressively increases with feed exposure time in water, and feed coating agent and pellet size significantly affect the leaching of medicated fish feed (Barreto et al., 2018). Additionally, fish farmers commonly include antibiotics in the transport water and use a 10 mg L^−1^ concentration of florfenicol to verify survival after transport (Klein et al., 2013).

The environmental risk assessment and ecotoxicological testing are useful methods for correlating laboratory toxicity data and predicting concentration conditions to determine appropriate concentrations to avoid damage in the environment (Zagatto and Bertoletti, 2008). Ecotoxicity studies about FF toxicity have been performed with pacu (LC (I) 50; 48h > 1000 mg L^−1^), along with *Oncorhynchus mykiss* (LC (I) 50; 48h > 780 mg L^− 1^) and *Lepomis macrochirus* (LC (I) 50; 48h > 830 mg L^−1^) (Schering-Plough animal health, 2009). Therefore, FF can be considered practically without toxicity to these species, however antibiotics are used indiscriminately and large quantities reach natural water resources. Currently, there is no information on quantity released or environmental impact, nor on the effects of florfenicol toxicity on the health of cultivated species exposed through the water. In this work, 11.72 mg L^−1^ of florfenicol was applied to juvenile Nile tilapia (*Oreochromis niloticus*) through the water to determine the muscle bioconcentration, hematological parameters and oxidative stress of these fish after 24 h and 48 h of exposure.

## 2 Material and methods

### 2.1 Experimental conditions

Juvenile Nile tilapia (*Oreochromis niloticus*) were obtained from a commercial fish farm (Polettini, Mogi Mirim, SP), acclimated to the experimental system and fed with commercial diet (Guabi® specific for tilapia: 4–5 mm; 10 % moisture; 32 % protein; 6.5 % fat) for three weeks. In this period, fish consumed an average of 4% of their body weight.

After the acclimation period, 160 fish were anesthetized with benzocaine (100 mg L^−1^), weighed individually (41.54 ± 1.09 g), and distributed randomly in 10 aquaria with 300 L of water in an individual system with controlled temperature and continuous aeration. The experimental design was completely randomized with two experimental groups: Control Group (Control, exposure free) and Florfenicol (FF) Group (exposure the concentration of 11.72 mg L^−1^) with five repetitions (16 fish per repetition/experimental unit).

Certified standards of florfenicol were acquired from Sigma Aldrich (St. Louis, MO, USA). Nile tilapia were exposed to water containing florfenicol at a nominal concentration of 10 mg L^−1^, which is equivalent to 1/100 of the LC50 (Carraschi et al., 2011; OECD, 2012), which is the dose that fish famers commonly use in transport water to guarantee survival after transport (Klein et al., 2013). Contaminated water from experimental aquariums were sampled (5 mL of water collected in the middle of the water column) for a quantitative description of florfenicol contamination. Therefore, the actual exposure concentration determined was 11.72 mg L^−1^ of florfenicol. According to the OECD (OECD, 2012), the concentration of the test substance in the chambers was maintained within ± 20% of the mean of the measured values during the uptake phase. The 11.72 mg L^−1^ value falls within the 20% range in relation to the nominal concentration of 10 mg L^−1^.

During the experimental period, the temperature (26 ± 1 °C), conductivity, (0.09 ± 0.01 μS cm^−1^), dissolved oxygen (8.1 ± 0.5 mg L^−1^) and pH (7.2 ± 0.2) were measured daily using a multiparameter - water quality checker (U-50, Horiba, Minami-ku, Kyoto, Japan). Total ammonia (0.31 mg L^−1^) was measured weekly using a commercial kit (Hach, Loveland, CO, USA). Aquariums were cleaned periodically by siphoning and renewing 20 % of the total water volume in the system. The values of water quality parameters were adequate for the species, according to Boyd et al. (1998).

Subsequently, tilapia was not feed for 24 h and exposed to FF for 48h. Two fish per experimental unit (total of 40 fish) were anesthetized with benzocaine (100 mg L^−1^) and used for blood and liver collections. The hematological and biochemical parameters of samples were analyzed 24 and 48 h after exposure.

### 2.2 Hematological parameters

Blood was collected by caudal puncture with a syringe containing EDTA anticoagulant (3%). To determine the percentage of hematocrit, blood was inserted into capillary tubes and centrifuged in a microhematocrit centrifuge (NI 1807 Nova Instruments, Piracicaba, SP, Brazil) for 5 min at 10,000 rpm. The analysis to quantify the hemoglobin concentration was performed using a cyanmethemoglobin kit (Labtest Diagnostica, Belo Horizonte, MG, Brazil). The blood was diluted in a citrate-formaldehyde solution (1: 200) and the erythrocyte count was performed in a Neubauer chamber. The mean corpuscular volume (MCV) and the mean corpuscular hemoglobin concentration (MCMC) were also calculated (Wintrobe 1934).

Differential and total leukocyte and total thrombocyte counts were performed on blood smears stained with May-Grunwald-Giemsa-Wright under a microscope with an oil immersion objective (100 x).

### 2.3 Obtaining tissue and determination of oxidative metabolism

After 96 h period of exposure to FF all the fish were submitted blood sampling and next up, they passed by process of euthanized by transection of the spinal cord to get the tissues. The liver collected from each fish was properly washed with saline water solution (0.9 % NaCl) to clean the excess of the dirty caused by the handling process. After that, the liver was dried with filter paper, identified and stored at - 80 °C for further biochemical analysis.

To determine oxidative metabolic the individual frozen liver samples were weighed and homogenized using the homogenizer of tissues Turratec TE 102 (Tecnal, SP, Brasil) in 1800 rpm by 1 min and buffer ice containing 0,1 M sodium phosphate pH 7,4. The homogenized was centrifuged at 12,000 rpm, 4 °C for 20 min in the refrigerated centrifuge Hermle-Z323K (Hermle Labortechnik, Germany) and the supernatant was used as the enzyme source. An aliquot of the samples were obtained to determine total liver protein according to the Bradford (1976) method, adapted for a Dynex MRXTC 250 microplate reader, as described by Kruger (1994).

To determine the formation of the lipid hydroperoxide an assay of the iron oxidation by xylenol orange (FOX) was used as described by Jiang et al. (1992). In the method the samples of tissue are previously deproteinized with TCA 10 %, then are added the reagents of FOX 250 μM ferrous ammonium sulfate, 100 μM orange xylene, 25 μM H2SO4, and 4 mM BHT in 90% (v/v) methanol. HP levels were detected spectrophotometrically at 560 nm, and presented as nmol. g tissue^−1^. The CAT activity was determined according to Aebi (1983). In this method was monitored the decrease in hydrogen peroxide (H_2_O_2_) every 15 s during 2 min, reading in absorbance of 240 nm. The data are showed UB/mg of protein ^−1^. The GST activity was measured according to Habig et al. (1974). The substrate l-chloro-2.4-dinitrobenzene (CDNB) was used for measurement of the enzyme activity. The activity of the GST was read in absorbance of 340 nm for 4 min with a peak of the read every 30 s. The data are shown in mmol.min^−1^.mg of protein ^−1^.

Spectrophotometric readings were made in a SpectronicGenesys 5 (Milton Roy Company, PA, USA) spectrophotometer, and the microplate readings were carried out with a Tecan-SNR reader (Sunrise-Basic Tecan, NS 1105003419 Groding, Austria).

### 2.4 Analytical method to determine florfenicol residues in water and tilapia muscle

Since the concentration of florfenicol added in the water was 11.72 μg mL^−1^, the water sample, was diluted by ten-fold by adding 10% of the water sample from the aquariums to 90% of the mobile phase. The samples were filtered at 0.22 μm and injected into the chromatographic system.

The analytical method used to determine florfenicol residues in tilapia muscle was performed according to Shiroma et al. (2019). The extraction was done by using the modified QuEChERS, with a tube with 1.00 g of the sample and 5 mL aliquot of acetonitrile containing 1.0% (v/v) acetic acid. The tube was vortexed for 1 min. Magnesium sulfate (2.0 g) and C_18_ (50 mg) were added, followed by vortexing for 1 min and centrifuging at 3000 × g for 5 min. A 1.0 mL aliquot of the supernatant was dried with nitrogen and the residue was resuspended in the mobile phase, filtered with a 0.22 μm nylon filter and injected without pH adjustment into the chromatographic system.

The quantification of FF in water samples and tilapia muscle was obtained by using a Liquid Chromatography coupled to a Mass Spectrometry (LC-MS/MS), and a Lichrocart Cartridge Purospher Star C8 HPLC column (250 mm × 4.6 mm, 5 μm particle size) was used for separation. The analytes were separated with a mobile phase consisting of Milli-Q water and acetonitrile, both with 0.1% formic acid. Isocratic elution was used, with a 40:60 (v/v) mobile phase. The flow rate was 0.50 mL min^−1^ and the injection volume was 10 μL. HPLC was interfaced to an IonTrap AmaZon X mass spectrometer from Bruker (Billerica, MA, USA) equipped with an electrospray ionization interface (ESI). Data acquisition was performed using QuantAnalysis 2.0 from Bruker Daltonics (Billerica, MA, USA) software. The recovery rate was 70–79%, matrix effect was -28%, limit of detection (LOD) was 0.0625 μg g^−1^ and limit of quantification (LOQ) was 0.125 μg g^−1^.

### 2.5 Statistical analysis

All data was tested for normality (Shapiro-Wilk) and the means were submitted to an analysis of variance (ANOVA). Means were compared by Tukey tests with p-value<0.05 to estimate the level of significance. To assess the hematological and hepatic oxidative stress response parameters, a completely randomized design with a factorial arrangement of 2 × 2, i.e., 2 treatments (control and FF) x 2 sampling times (24 and 48 h post-exposure), was set up.

## 3 Results

The FF dose administered to fish through the water was 11.72 μg mL^−1^. No FF residues were detected in the fish muscle of the control group. The maximum FF residues detected at 24 and 48 h in the florfenicol-exposed group were 0.38 and 0.92 μg g^−1^, respectively (Table 1).

**Table 1:** Fish muscle concentrations of florfenicol (FF; μg g^−1^) from each tank at 24h and 48h exposure times.

Time	Control	FF tank (μg g^−1^)				
		1	2	3	4	5
24 h	nd	0.38	0.36	0.33	0.30	0.29
48 h	nd	0.55	0.49	0.92	0.68	0.51

nd = not detected.

The general health conditions of the fish were normal during the experiment, with no mortality in any experimental group during the 48 h exposure period.

There were no interactions between treatments exposed to water with (FF) or without (control) florfenicol and sampling times (24 and 48 h after exposure) for the number of erythrocytes, hemoglobin, hematocrit, MCHC, MCV, TPP, leukocytes, and thrombocytes.

In this study, there were no differences in the number of erythrocytes, hemoglobin, hematocrit, MCHC, MCV, TPP, leukocytes, and thrombocytes between the control and FF groups (Table 2), regardless of the sampling time (P > 0.05) or exposure (P > 0.05).

**Table 2:** Mean ± SD (n = 10) of erythrocyte number, hemoglobin concentrations, hematocrit, mean corpuscular hemoglobin concentration (MCHC), mean corpuscular volume (MCV), leukocytes and thrombocytes of Nile tilapia in the water with (FF) or without (control) florfenicol.

Treatment	Control		FF	
Time	24 h	48 h	24 h	48 h
Erytrocytes (x 10^6^ μL)	1.60 ± 0.19	1.66 ± 0.24	1.65 ± 0.26	1.72 ± 0.28
Hemoglobin (g dL^−1^)	8.43 ± 1.19	7.92 ± 1.55	8.99 ± 0.52	8.17 ± 1.04
Hematocrit (%)	30.33 ± 4.03	28.70 ± 3.74	31.83 ± 3.84	30.40 ± 4.09
MCHC (g dL^−1^)	28.67 ± 3.93	26.95 ± 6.60	28.64 ± 3.25	24.99 ± 4.73
MCV (fL)	188.23 ± 38.07	217.88 ± 34.21	183.24 ± 47.27	203.35 ± 48.10
TPP (mg dL^−1^)	4.33 ± 0.09	4.47 ± 0.04	4.50 ± 0.04	4.02 ± 0.04
Leukocytes (μL^−1^)	114117 ± 4497	98495 ± 35981	92797 ± 46671	104287 ± 48327
Trhombocytes (μL^−1^)	43849 ± 17080	36909 ± 11358	50892 ± 18475	43675 ± 21845

**Figure 1:**
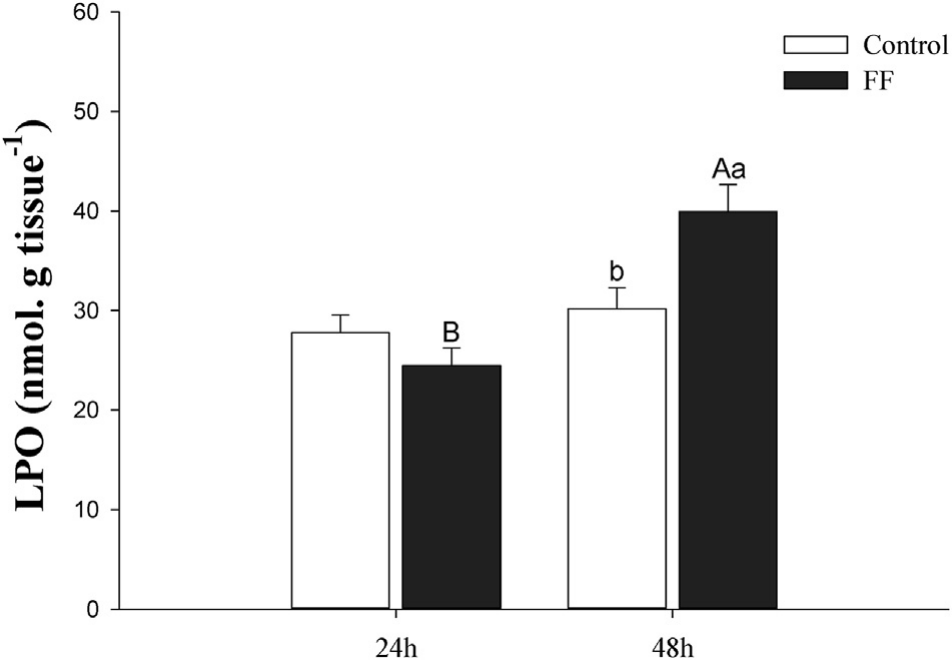
Levels of lipid peroxidation (LPO; nmol g tissue^−1^) in the liver of Nile tilapia in the water with (FF) or without (Control) florfenicol, 24 and 48h after exposure. Values are presented as the mean ± standard deviation (N = 10). Means followed by asterisks are significantly different (P < 0.05).

There were interactions between treatments exposed to water with (FF) or without (control) florfenicol and sampling times (24 and 48h after exposure) for LPO levels. FF at 11.72 mg L^−1^ increased lipid peroxidation levels within 48h after exposure (Figure 1). Comparing the levels of lipid peroxidation observed at 24h and 48h after exposure, they showed no differences in the control goup, while the group exposed to 11.72 mg L^−1^ of FF showed the highest value at 48h after exposure (P < 0 .05).

**Figure 2:**
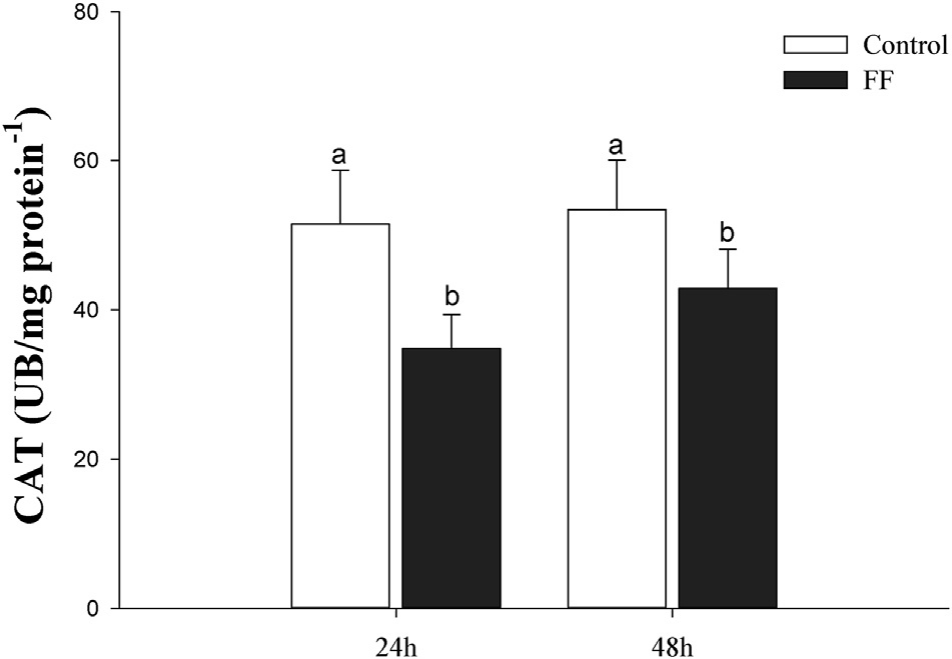
Catalase activity (CAT; UB mg protein^−1^) in the liver of Nile tilapia in the water with (FF) or without (Control) florfenicol, 24 and 48h after exposure. Values are presented as the mean ± standard deviation (N = 10). Small letters compare grouped sampling times in each treatment and capital letters compare grouped treatments at each sampling time (P < 0.05). Small letters compare grouped sampling times in each treatment, and capital letters compare groups in each sampling time for p < 0.05.

**Figure 3:**
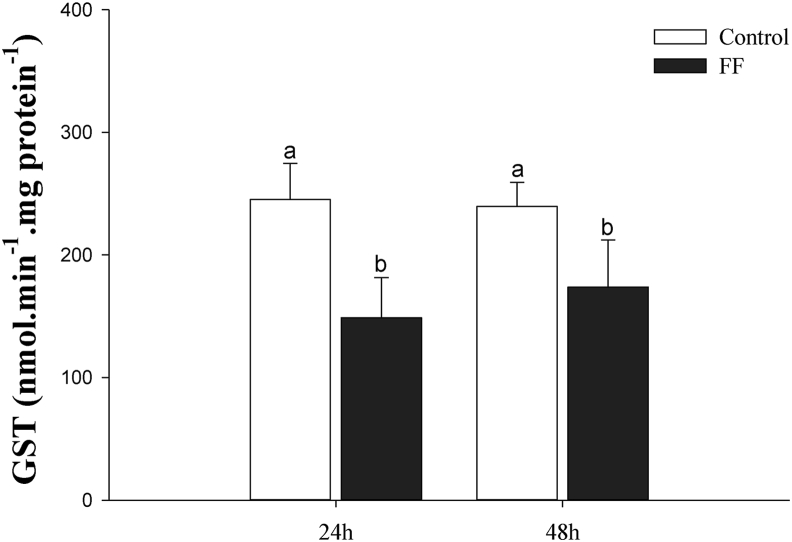
Glutathione S-transferase activity (GST; nmol min^−1^ mg protein^−1^) in the liver of Nile tilapia in the water with (FF) or without (Control) florfenicol, 24 and 48h after exposure. Values are presented as the mean ± standard deviation (N = 10). Small letters compare grouped sampling times in each treatment and capital letters compare grouped treatments at each sampling time (P < 0.05).

There were no interactions between treatments (control and exposed) and sampling times (24 and 48 h after exposure) for catalase (CAT) and glutathione S-transferase (GST) activity. The CAT (Figure 2) and GST (Figure 3) in the group exposed to FF were higher compared to the control group, regardless of the sampling time (P < 0.05). Regardless of treatments, CAT and GST were not different at 24 and 48h after exposure (P > 0.05).

## 4 Discussion

In this study, we investigated whether the presence of the veterinary antibiotic Florfenicol in cultivation water could induce changes in the health parameters of Nile tilapia. The results found herein indicate that Nile tilapia exposed to florfenicol at a concentration of 11.72 mg L^−1^ for 48 h is bioaccumulated in tissues, did not show changes in hematological parameters, but caused oxidative cell damage. Florfenicol is a veterinary medicine widely used in fish farming, however, inappropriate use of antimicrobial drugs can cause treatment failure, development of bacterial resistance, violation of drug residue limits, toxicity to fish and environmental pollution (Rairat et al., 2019).

Florfenicol is used in Brazilian aquaculture to treat bacterial diseases, such as *Aeromonas* sp. or *Streptococcus agalactiae*, in Tilapia species and their hybrids (SINDAN, 2017). Sick animals take a longer time to eat food, which increases the amount of time that medicated foods remain in waters and facilitates FF leaching; therefore, the constant use of medicated feed in fish farms leads to high levels of FF in the water (Barreto et al., 2018). As the antibiotic tested herein has wide therapeutic applications in psiculture, florfenicol has been frequently detected in natural aquatic ecosystems (Jiang et al., 2011; Yan et al., 2013; Zhao et al., 2015), at concentrations as high as 11 mg L^−1^ (Zong et al., 2010). In this sense, it is important to assess whether this concentration can affect aquatic organisms that live in nearby waters and the potential damages to the aquatic ecosystem. Therefore, hematology and biomarkers of oxidative stress are good indicators ofthe toxicity of environmental concentrations of florfenicol on tilapia health. Non-target aquatic species have also been studied to assess environmental toxicology. Zhang et al. (2019) investigated the long-term (21-d) influence on the reproduction and growth of and the acute (24-h) oxidative response and tissue damage in the crustacean *Daphnia magna* after exposure to phenicol drugs, and found that florfenicol exposure likely caused more adverse effects than chloramphenicol and thiamphenicol.

Hematological variables are often used to detect physiological changes and can be an essential index for the general health status of several fish species (Ivanc et al., 2005; Satake et al., 2009), helping identify the adverse situations that fish are exposed to (Fazio, 2019). Hematocrit, hemoglobin, red blood cell count and hematimetry indices, as MCV and MCHC, total plasma protein, leukocytes and total thrombocytes were not altered by acute exposure to FF. All blood parameters analyzed in this study were within the normal range reported for Nile tilapia in a semi-intensive fish farming system (Bittencourt et al., 2003; Tavares Dias 2015). These results agree with studies that assessed acute toxicity and the risk of intoxication through lethal concentration and showed that FF is without toxicity for fish such as pacu (LC (I) 50; 48h > 1000 mg L^−1^) (Carraschi et al., 2011), *O. mykiss* (LC (I) 50; 48h > 780 mg L^−1^) and *L. macrochirus* (LC (I) 50; 48h > 830 mg L^−1^) (Schering-Plough animal health, 2009). However, Botelho et al. (2015) investigated the effects of environmental concentrations of florfenicol on the erythrocyte genetic material in tilapia juveniles (*Oreochromis niloticus*) and found that low environmental concentrations of florfenicol (425 ng L^−1^) are genotoxic to erythrocytes and damage the DNA molecule.

Our findings show that FF at a concentration of 11.72 mg L^−1^ is rapidly absorbed from water in a single episode and manifests changes in the hepatic ROS markers of *O. niloticus*, revealed by the levels of lipid peroxidation, catalase activity and S- glutathione transferase. Such scenario demonstrates the potential of environmental FF to induce oxidative stress in hepatocytes of this species after a short exposure time.

Analyzing the oxidative stress markers, there was a significant increase in the values of LPO and a reduction in the activity of the antioxidant enzymes CAT and GST, meaning that the critical balance between oxidant and antioxidant was disrupted when the animals were exposed to FF (11 mg L^−1^) due to depletion of antioxidants and excessive accumulation of ROS in hepatocytes. When this event occurs in an organism, excess ROS is continuously generated as a by-product of metabolic processes such as superoxide anion (O^2-^), and hydrogen peroxide (H_2_O_2_) and hydroxyl radical (OH) can interact with macromolecular biological agents, producing enzymatic inactivation, lipid peroxidation and DNA damage (Giordano et al., 2009; Kurutas, 2015; Nita and Grzybowski, 2016).

LPO is an indicator of oxidative damage to cellular components and is the primary step in the cytotoxic event that triggers a sequence of lesions to cell membranes and lipoproteins (Lima and Abdalla, 2001). In ecotoxicological studies of aquatic organisms, LPO has been widely used to indicate oxidative stress in different fish species exposed to a variety of xenobiotics present in water and the liver is the main organ analyzed. Herein, the LPO values significantly increased in the after 48 h of exposure, revealing that FF could have an oxidizing action in the hepatic tissue of Nile tilapia. This can cause oxidative stress and, consequently, hepatotoxic effects that can lead to sequelae or mortality of fish.

The enzymes with antioxidant functions are potential indicators of oxidative stress, however, the activity and intensity of these enzymes (increase or inhibition) in the cells responds to the nature of the chemical stressor, concentration tested (Sun et al., 2006; Javed et al., 2016), species studied and respective fish life stage. In order to minimize the damage of ROS (Reactive oxygen species) in an organism, the CAT enzyme acts in the first line of defense of antioxidants, responsible for the neutralization and reduction of H_2_O_2_ peroxide in O_2_ and H_2_O in the peroxisome (Oruç and Üner, 2000; Coelho et al., 2011). GST integrates a class of multifunctional enzymes involved in the detoxification of a wide variety of xenobiotics with an electrophilic center, thus protects the cell from oxidative stress (Rao, 2006; Huber et al., 2008).

In our study, CAT activity showed significantly reduced in liver tissue after 24 and 48 h of exposure to FF. Both the reduction and increase in CAT activity indicates impairment in the normal oxidation process, suggesting flaws in the antioxidant defense system (Clasen et al., 2018). The reduced CAT activity may be due to inactivation by the superperoxic free radical or by excess H_2_O_2_ production (Vutukuru et al., 2006). In addition to enzyme inactivation, the accumulation of H_2_O_2_ can lead to the formation of hydroxyl radicals, which is more reactive in the biological system (Sun et al., 2006). Thus, we can consider that the reduction of CAT activity in the presence of FF at a concentration of 11 mg L^−1^ left the fish more susceptible to several pathologies linked to oxidative stress, promoting tissue damage and compromising the functioning of the fish's liver tissue through the accumulation of O^2-^ or H_2_O_2_

GST also showed reduced activity when exposed to FF in at 24 and 48 h. This enzyme participates in an important integrated antioxidant defense system of phase II in the enzymatic detoxification process of xenobiotics (Carletti et al., 2008). In phase II, enzymes such as GST catalyze the conjugation of xenobiotics (or phase I metabolites), making them easily excreted due to their high water solubility (Huber et al., 2008; Clasen et al., 2018). In this sense, the high activity of GST may reflect better protection against the toxicity of xenobiotics and, consequently, the formation of radicals in the biological system (Rao, 2006). Nevertheless, the reduced activity of this enzyme reported in our study indicates damage to the system in phase II of the FF detoxification process, making it difficult for fish to eliminate FF from their bodies. Elia et al. (2016) evaluated the effect of FF on Rainbow Trout *Oncorhynchus mykiss* treated for 10 d with 7.5 and 15 mg/kg FF followed by a withdrawal period of 5 d,and detected oxidative damage during the antibiotic treatment as a consequence of the effect of FF toxicity and a rise in total glutathione and glutathione S-transferase levels, even after the withdrawal period.

In conclusion, the acute exposure to FF content in the cultivation water did not alter the hematological variables, but did cause oxidative cellular damage in tilapia. Therefore, our results suggest that florfenicol antibiotics could cause toxicity in organisms and, thus, aquatic ecosystems, even at relatively low environmental concentrations in relation to the LC50-48h that has been reported for fish species.

Our results help describe the potential ecological risks of this antibiotic and other pharmaceutical products in aquatic environments and can be used to develop management strategies to reduce these compounds in aquatic systems in order to limit their impacts on the lives of free-living fish and other aquatic animals. In addition, this study helps spread awareness about the intensive use of these compounds to minimize environmental risks, contamination and bacterial resistance, as well encourage the use of alternative products, such as immunostimulants, extracts and essential oils from plants, to fight diseases in psiculture.

## Declarations

### Author contribution statement

L.S. Shiroma, M.P. Soares and I.L. Cardoso: Conceived and designed the experiments; Performed the experiments; Analyzed and interpreted the data; Wrote the paper.

M.M. Ishikawa, S.C.N. Queiroz and C.M. Jonsson: Conceived and designed the experiments; Analyzed and interpreted the data; Contributed reagents, materials, analysis tools or data; Wrote the paper.

### Funding statement

This work was supported by National Bank for Economic and Social Development (BNDES) and 10.13039/501100003046Brazilian Agricultural Research Corporation (Embrapa), Brazil (0117020010606007). This study was financed in part by the 10.13039/501100002322Coordination for the Improvement of Higher Education Personnel (CAPES)/Brazil – Finance Code 001.

### Data availability statement

Data included in article/supplementary material/referenced in article.

### Declaration of interests statement

The authors declare no conflict of interest.

### Additional information

No additional information is available for this paper.
